# Endoscopic Balloon Dilation of Gastric Stenosis secondary to Polyarteritis Nodosa and Arterial Thrombosis in an Adolescent

**DOI:** 10.1097/PG9.0000000000000198

**Published:** 2022-04-08

**Authors:** Rubén Peña-Vélez, Mariana Roldán-Montijo, Sharon Imbett-Yepez, Jaime Ramírez-Mayans, Alejandro Loredo-Mayer, Ericka Montijo-Barrios

**Affiliations:** From the * Department of Pediatric Gastroenterology and Nutrition, Instituto Nacional de Pediatría. Mexico City, Mexico; † School of Medicine, Universidad Nacional Autónoma de México, Mexico City, Mexico.

**Keywords:** pediatric, endoscopy, therapeutic endoscopy, spontaneous gastric perforation

## Abstract

A 13-year-old female with polyarteritis nodosa underwent a partial gastrectomy for ischemic necrosis and gastric perforation following left gastric artery thrombosis. She later presented with vomiting, early satiety, weight loss, and severe malnutrition, when she was diagnosed with an occlusive gastric stricture. She successfully underwent repeated therapeutic endoscopic balloon dilations until the endpoint of 15–18 mm lumen was achieved without any complications, and her symptoms resolved.

## INTRODUCTION

Gastric stenosis is a rare entity in children. It occurs more frequently due to congenital hypertrophic pyloric stenosis in infants or from caustic ingestion in children.^1^ We report a child with spontaneous gastric perforation from ischemic necrosis following left gastric artery thrombosis due to polyarteritis nodosa (PAN). Following partial gastrectomy, the patient subsequently developed severe gastric stenosis, which responded to serial therapeutic endoscopic balloon dilations.

## CASE REPORT

A 13-year-old female, with no prior history of gastrointestinal or systemic symptoms, presented to the emergency department with severe abdominal pain and vomiting for 24 hours. Due to signs of acute abdomen, an exploratory laparotomy was performed. It revealed a minor curvature gastric perforation, and necrotic fundus and gastric body. Consequently, a 60% partial gastrectomy and gastrostomy were performed. She spent two months in the Intensive Care Unit. A diagnosis of PAN was confirmed according to the 2008 Ankara consensus,^2^ as she fulfilled clinical (impaired kidney function), arteriographic (abdominal angiogram with irregularities in the caliber of the gastric arteries) and histologic criteria (evidence of necrotizing vasculitis in medium-caliber arteries on stomach biopsy). During this hospitalization, nutritional support was managed by a mixed technique, with enteral nutrition by gastrostomy tube for the most part, and, to a lesser extent, oral feeding. After stabilization and initiation of the immunosuppressive treatment of the PAN, the patient was discharged.

**FIGURE 1.**
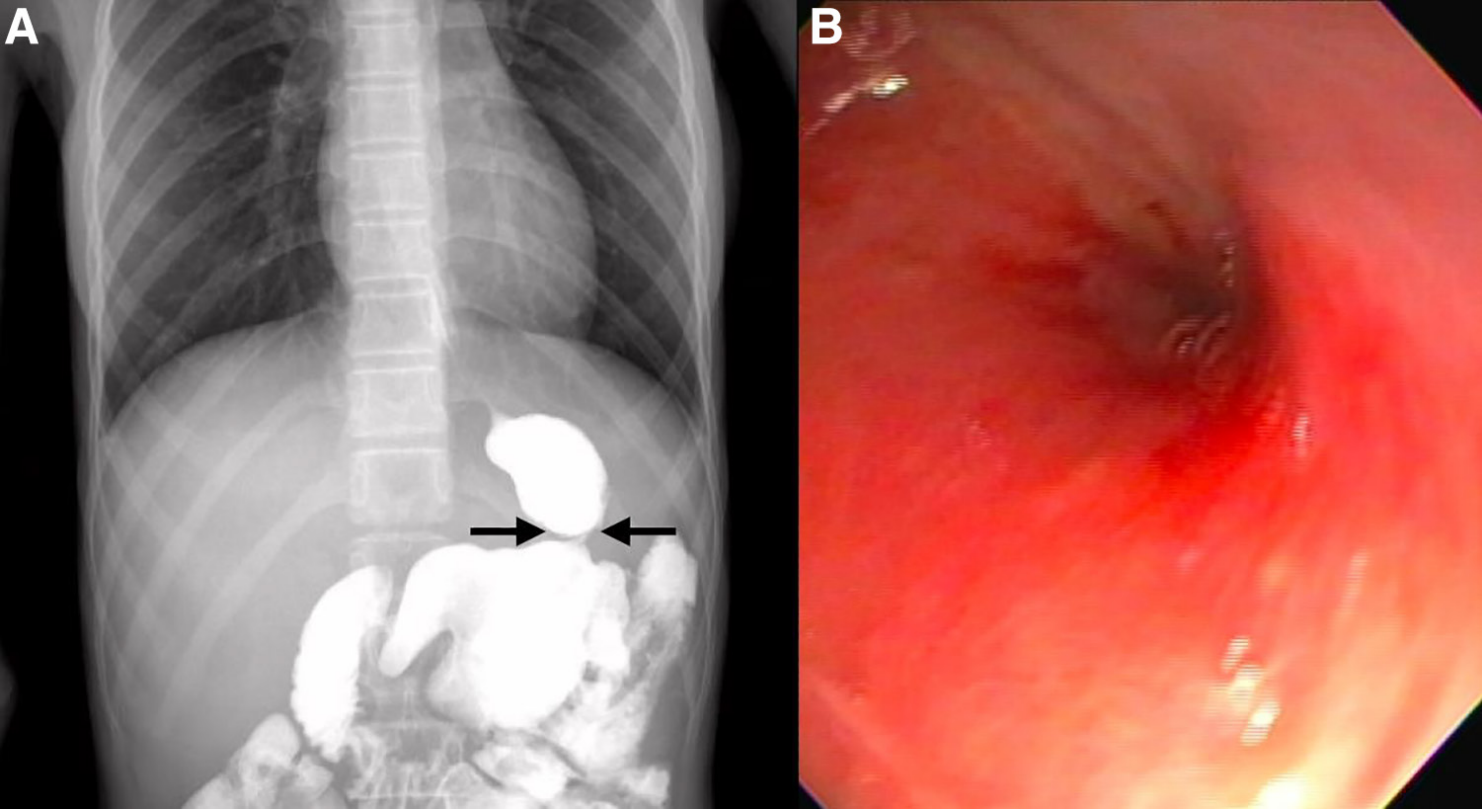
Gastric stenosis. A) Contrast gastrography showing the site of stenosis. B) Gastric endoscopy showing punctate stenosis (<5 mm).

Four months after the gastrectomy, the patient developed multiple, nonbilious, random and postprandial vomiting episodes and early satiety which caused significant weight loss and, consequently, severe malnutrition (weight: 21.4 kg, length: 148.5 cm, BMI Z-score: −8.93) Abdominal examination showed a scaphoid abdomen with normal peristalsis, and no tenderness or hepatosplenomegaly. She was admitted to the Gastroenterology ward where an upper GI series and upper digestive endoscopy were performed, documenting a 5-mm gastric body stenosis (Fig. 1). Nutritional support with continuous enteral feeding through the gastrostomy was initiated.

**FIGURE 2.**
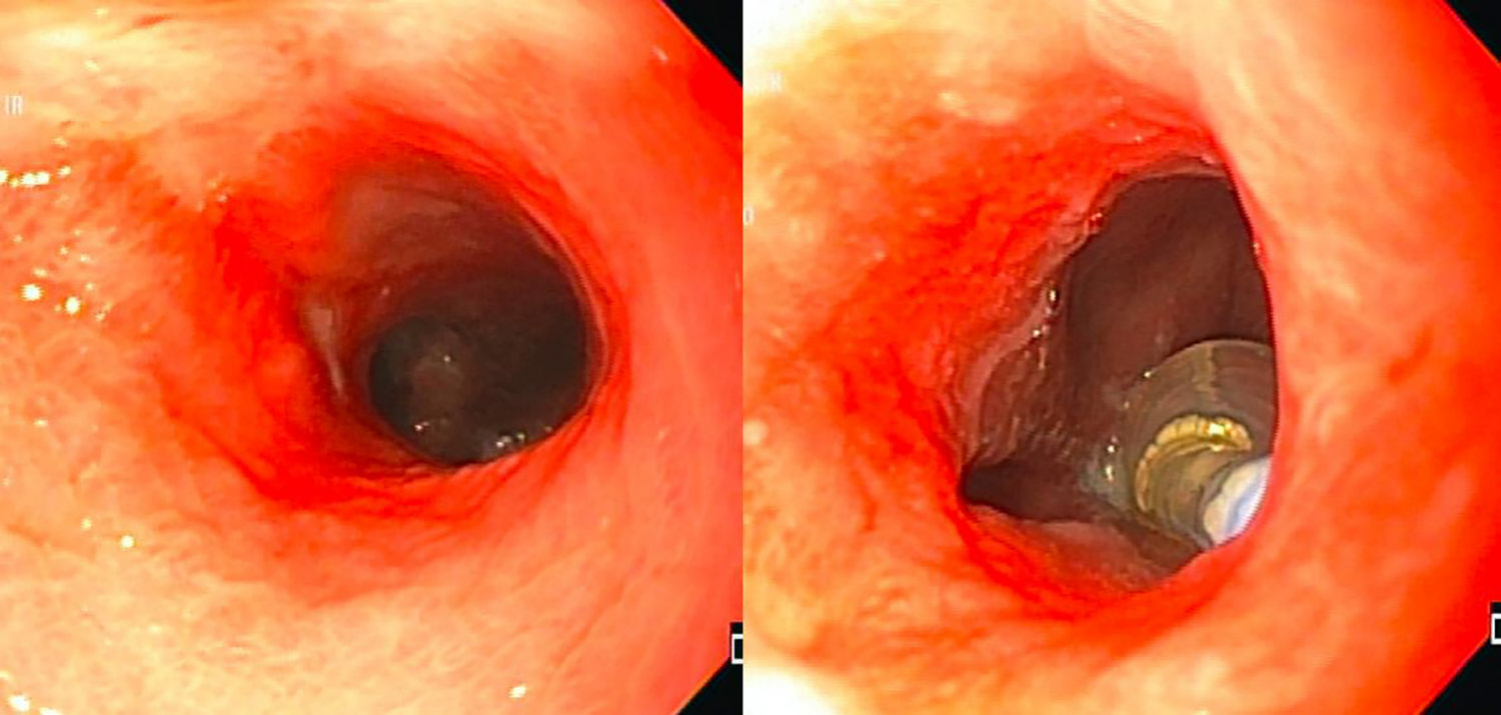
Endoscopy control after hydropneumatics dilation of the stenosis.

The first endoscopic dilation was performed with 8-9-10 mm hydropneumatic balloons (CRE PRO Wireguided Balloon Dilatation Catheters. Boston Scientific; Marlborough MA, EUA), at 60-second intervals. Thereafter, enteral feeding was steadily advanced to bolus feeds. One week later, a control endoscopy was performed, documenting an increase in the diameter of the stenosis to 12 mm, which allowed the passage of the endoscope (9.9 mm). A second dilation was done with 15-16-18 mm hydropneumatic balloons. After this, the volume of enteral and oral feeds increased, with adequate tolerance. During the third endoscopy, a 16-mm stenosis was observed, and dilation was made with an 18 mm hydropneumatic balloon (Fig. 2). No medications were injected into the stricture at any session.

Three months after the first endoscopic dilation, the patient remained asymptomatic. The oral and enteral feeds provided adequate caloric intake, and she had a satisfactory nutritional recovery (weight: 28 kg, length: 149.5 cm, BMI Z-score: −4.27). Since the site of the gastric stenosis was appropriately identified and a good response to the treatment with endoscopic dilatation was achieved, further imaging studies were not considered. The patient is currently on hydroxychloroquine, methotrexate, acetylsalicylic acid, and acenocoumarin.

## DISCUSSION

To our knowledge, spontaneous gastric perforation from PAN has not been reported in children. While perforation is reported from other areas of the gastrointestinal tract, only one case of gastric perforation secondary to PAN is documented in an adult, a 51-year-old male with a history of weight loss and abdominal pain with signs of acute peritonitis and free air in the abdominal cavity in a radiological study due to gastric perforation that required surgical management.^3^

Our case presents a child who developed significant gastric body stenosis after undergoing a partial gastrectomy for gastric necrosis and perforation associated with an autoimmune, medium vessel vasculitis. Acquired gastric strictures are uncommon in pediatrics. The usual form of presentation is nausea, vomiting, epigastric pain, early satiety, abdominal distention, abdominal mass, visible peristalsis, weight loss, and electrolyte imbalances.^4^ Our patient presented with multiple events of vomiting and early satiety leading to severe malnutrition.

In children, the common causes of acquired gastric outlet obstruction are peptic ulcer disease, ingestion of caustic substances, and surgical anastomoses. Less commonly, upper gastrointestinal tract involvement in Crohn’s disease and other rare conditions, including nonsteroidal anti-inflammatory drugs, infections (tuberculosis, cytomegalovirus), or iatrogenic, can result in gastric strictures.^5^ Gastric stenosis developed in our patient following gastrectomy performed for ischemic gastric necrosis.

With the recent advances in therapeutic upper digestive endoscopy, most reports on endoscopic balloon dilation of acquired gastric stenoses are from the adult population.^6^ This technique is less frequently needed or performed in the pediatric population.

Endoscopic balloon dilation is a relatively safe procedure with infrequent complications. Perforation and bleeding are rarely reported after balloon dilation.^5^ In our patient, dilation was performed up to 18 mm with no complications. A retrospective cohort study by Solt et al ^7^ found good response in 18 patients with postoperative strictures and in 6 patients with postvagotomy functional stenoses. Balloon dilation guided by fluoroscopy has also been similarly successful with response rates as high as 94%.^8^

Although a few authors report on endoscopic treatments for gastric stenosis,^9,10^ few publications report on endoscopic balloon dilation in children. This report aims to highlight the satisfactory outcome following endoscopic dilation, with adequate nutritional recovery and resolution of symptoms and no complications.
